# The adverse events of toripalimab in nasopharyngeal carcinoma based on FAERS database and bibliometric analysis

**DOI:** 10.1371/journal.pone.0326216

**Published:** 2025-06-20

**Authors:** Qian Guo, Dong Dong, Zihan Dang, Shuman Huang, Xinjie Qiao, Baiquan Zhang, Yulin Zhao

**Affiliations:** 1 Department of Rhinology, The First Affiliated Hospital of Zhengzhou University, Zhengzhou, China; 2 Department of Health Studies and Applied Educational Psychology, Columbia University, New York, New York, United States of America; 3 Department of Respiratory Medicine, The First Affiliated Hospital of Zhengzhou University, Zhengzhou, China; Peking University First Hospital, CHINA

## Abstract

**Background:**

Toripalimab, a monoclonal antibody designed to target PD-1, has recently received approval from the U.S. Food and Drug Administration (FDA) for use as a first-line treatment in adults diagnosed with metastatic or recurrent locally advanced nasopharyngeal carcinoma (NPC). The purpose of this study is to utilize the FAERS database and bibliometric analysis to examine adverse events associated with toripalimab in real-world settings, thereby enhancing the safety management of clinical medications.

**Methods:**

This research implemented a disproportionality analysis to assess the safety of toripalimab by reviewing all adverse event reports from the FAERS database dating back to 2004, wherein toripalimab was recognized as the main suspected medication. Various statistical techniques were applied in the analysis, such as the reporting odds ratio (ROR), proportional reporting ratio (PRR), multi-item gamma Poisson shrinker (MGPS), and Bayesian confidence propagation neural network (BCPNN), to evaluate the adverse events linked to toripalimab. CiteSpace is utilized to search for authors, countries, keywords, and various indicators within research fields, facilitating the identification of research hotspots and future trends.

**Results:**

From 2004 to 2024, 441 AEs linked to toripalimab were recorded across 27 SOCs. The top five SOCs were procedural complications, investigations, blood/lymphatic disorders, gastrointestinal disorders, and skin/subcutaneous disorders. At the PT level, the top five AEs by ROR were myelosuppression (n = 192, ROR 687.41), decreased granulocyte count (n = 11, ROR 515.72), immune-mediated hepatic disorder (n = 7, ROR 343.20), immune-mediated myocarditis (n = 3, ROR 214.68), and bicytopenia (n = 3, ROR 117.49). Additionally, 91.62% of AEs occurred within the first 30 days, and immune-related AEs were highlighted in bibliometric analysis.

**Conclusion:**

This research provides initial safety information regarding the real-world application of toripalimab, affirming previously acknowledged adverse effects and concurrently uncovering new possible risks. These results could act as important cautionary evidence for healthcare professionals and pharmacists engaged in administering toripalimab for NPC.

## Introduction

Nasopharyngeal carcinoma (NPC) is a malignant tumor that originates from the nasopharyngeal epithelium, exhibiting significant geographical disparities in its incidence rates. It is relatively common in regions such as North Africa, Southeast Asia, and Southern China, while being rare in Western countries, including Europe and the Americas [1,2]. Globally, NPC accounts for 0.7% of all new cancer cases and 0.8% of all cancer-related deaths [3]. Despite being highly sensitive to radiotherapy and chemotherapy, which leads to a favorable prognosis for most patients, approximately 15−30% of patients are at risk of local recurrence or distant metastasis after curative treatment. The median overall survival for these patients is about 20 months, highlighting the challenges in treating recurrent or metastatic NPC (R/M NPC) [4]. PD-1 is a crucial immune inhibitory molecule predominantly found on T cells, B cells, and natural killer cells [5]. Its ligand, PD-L1, is expressed on various cell types, including epithelial cells and immune cells, and interacts with PD-1 to suppress T cell function and induce apoptosis, thereby enabling tumor cells to evade immune surveillance and elimination. PD-1 inhibitors, known for their unique mechanism of action and significant efficacy against various malignancies, have garnered considerable attention in the field of cancer immunotherapy [6].

As NPC becomes more prevalent globally, this condition considerably diminishes patients’ quality of life and imposes a substantial burden on the healthcare system [7,8]. Toripalimab is a humanized anti-PD-1 antibody that has been approved by the FDA for the first-line treatment of nasopharyngeal carcinoma in combination with chemotherapy [9,10]. It functions by binding to PD-1 and inhibiting its interaction with the ligand. The interaction between toripalimab and PD-1 primarily involves the heavy chain of toripalimab and the FG loop of PD-1 [11,12]. Toripalimab has demonstrated effectiveness both as a monotherapy and in combination with radiotherapy for the treatment of patients with NPC. Its efficacy has been validated through numerous phase II and phase III clinical trials, which indicate significant improvements in the objective response rate (ORR), progression-free survival (PFS), and overall survival (OS) [13–15]. Despite the widespread use of toripalimab in clinical practice, there is limited information available regarding adverse events (AEs) associated with its treatment, primarily derived from clinical trials. According to the safety analysis of previous clinical trial reports and the description of toripalimab, the most common AEs include nausea, asthenia, hypothyroidism, pneumonia, pyrexia, vomiting, and decreased appetite [16].

The U.S. Food and Drug Administration (FDA) Adverse Event Reporting System (FAERS) database is the largest open pharmacovigilance database in the world, providing comprehensive details on all drugs marketed in the United States, along with extensive demographic information about users. Unlike the adverse event (AE) literature available in other databases such as PubMed, EMBASE, and MEDLINE, AEs in the FAERS database are recorded and analyzed individually, which enhances the fundamental nature of the data. This database is continuously updated and is publicly accessible through the official website of the U.S. Food and Drug Administration, facilitating the detection of emerging adverse event signals. Numerous studies have utilized this database to investigate adverse events associated with the clinical use of drugs [17,18]. Previous studies have made significant contributions to the development of the discipline through bibliometrics [19–21]. Consequently, we employed FAERS in conjunction with bibliometrics to conduct a comprehensive analysis and exploration of adverse reactions to toripalimab, thereby providing new insights for clinical practitioners and pharmacists.

## Materials and methods

### Data sources, management, and study design

**Fig 1:**
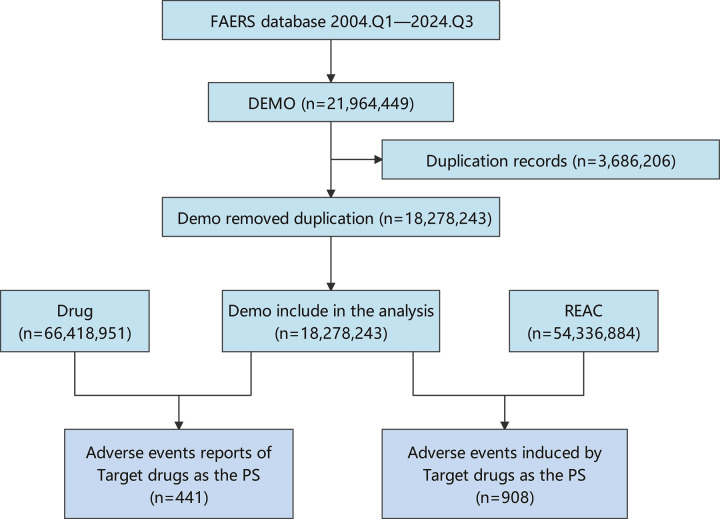
Flow diagram for the selection of AEs associated with toripalimab from FAERS database.

This study utilized raw data from the FAERS database, a publicly accessible, voluntary reporting system primarily populated by consumers, health professionals, pharmacists, physicians, and other stakeholders [22]. The assessment encompasses all adverse event reports presented as raw ASCII packets, identifying Toripalimab as the primary drug of interest, covering the period from the first quarter of 2004 to the third quarter of 2024. Within the data management framework, the processes of record deduplication and the standardization of AE terminology were implemented. In the initial phase, duplicate reports were removed based on methodologies outlined by the U.S. FDA. Specifically, reports were sorted by case identifier (CASEID), the date of FDA receipt (FDA_DT), and each report’s unique identifier (PRIMARYID). For those reports that shared the same CASEID, the one with the latest FDA_DT was retained. In cases where both CASEID and FDA_DT were identical, the report with the highest PRIMARYID was kept. Subsequently, beginning in the first quarter of 2019, deduplicated reports were eliminated in accordance with the CASEID values found in the deleted report catalog, each quarterly data package containing such documentation. Additionally, the most recent version of the Medical Dictionary for Regulatory Activities (MedDRA 27.1) was utilized to classify adverse event terms in the FAERS database, which included preferred terms (PT) and their corresponding system organ class (SOC). These updates were subsequently employed for further analysis. A comprehensive flowchart depicting the study design is available in Fig 1. All bibliometric analyses discussed in this paper were retrieved from the Web of Science, covering the search period from 2019 to 2025. This period marks a significant increase in scholarly research on the use of Toripalimab for the treatment of nasopharyngeal carcinoma, particularly with advancements in causal inference methodologies. The search query (Toripalimab OR PD1 inhibitor OR Anti-PD1 therapy) AND (nasopharyngeal carcinoma OR nasopharyngeal cancer OR nasopharyngeal tumor OR nasopharyngeal malignancy OR nasopharyngeal neoplasm) was input into CiteSpace for statistical analysis. Following the initial search, a rigorous screening of the studies was conducted, which included peer-reviewed articles on the treatment of nasopharyngeal carcinoma with Toripalimab and excluded irrelevant articles.

### Statistical analysis

A detailed analysis was conducted to summarize the features of the adverse event reports related to toripalimab. To detect potential signals of adverse effects, four methods of disproportionality analysis were utilized: reporting odds ratio (ROR) [23], proportional reporting ratio (PRR) [24], Bayesian confidence propagation neural network (BCPNN) [25], and multi-item gamma Poisson shrinker (MGPS) [26]. The criteria utilized for signal detection in the PRR approach were founded on the thresholds established by the joint standard method of the Medicines and Healthcare Products Regulatory Agency (MHRA). AEs were categorized as potential adverse reactions if they exceeded the positivity threshold in at least one of the analytical methods employed. A comprehensive two-by-two contingency table is provided in S1 Table, while the relevant formulas and thresholds for executing the disproportionality analysis can be found in S2 Table. The time to onset of these adverse events was determined by the interval between the documented occurrence of the toripalimab-associated adverse event and the initiation of treatment. All analyses were performed using SAS software, version 9.4.

### Ethics statement

Ethical approval was not required for the study involving humans in accordance with the local legislation and institutional requirements. Written informed consent to participate in this study was not required from the participants or the participants’ legal guardians/next of kin in accordance with the national legislation and the institutional requirements.

## Results

### Descriptive analysis

This research encompassed 441 individuals within the intended drug demographic, during which 908 adverse events were noted. Toripalimab emerged as the primary suspected medication (Fig 1). Of the reports, 97.05% were categorized as Not Specified, predominantly submitted by pharmacists (89.57%). The severe cases constituted 99.77%, while non-severe instances made up 0.23%. The overwhelming majority of reports were sourced from China (99.55%), with additional information available in Table 1.

**Table 1:** Characteristics of AEs reports.

Characteristics	n (%)
Sex	
Female	5 (1.13)
Male	8 (1.81)
Not Specified	428 (97.05)
Age (years)	
< 18	0 (0.00)
18–44	1 (0.23)
45–64	5 (1.13)
≥ 65	7 (1.59)
Not Specified	428 (97.05)
Age (years)	
N (Missing)	13 (428)
Mean (SD)	62.38 (9.10)
Median (Q1,Q3)	65.00 (58.00,69.00)
Min,Max	41.00,74.00
Report year	
2023	61 (13.83)
2024	380 (86.17)
Reporter	
Consumer	6 (1.36)
Pharmacist	395 (89.57)
Physician	40 (9.07)
Serious Report	
Serious	440 (99.77)
Non-Serious	1 (0.23)
Outcomes	
Life-Threatening	57 (12.93)
Hospitalization – Initial or Prolonged	274 (62.13)
Disability	18 (4.08)
Death	9 (2.04)
Congenital Anomaly	1 (0.23)
Required Intervention to Prevent Permanent Impairment/Damage	0 (0.00)
Other	134 (30.39)
Adverse event occurrence time (days)	
0–30d	361 (81.86)
31–60d	7 (1.59)
61–90d	6 (1.36)
91–120d	6 (1.36)
121–150d	5 (1.13)
151–180d	0 (0.00)
181–360d	5 (1.13)
>360d	4 (0.91)
Missing or outlier (less than 0) (%)	47 (10.66)
Adverse event occurrence time (days)	
N (Missing)	394 (47)
Mean (SD)	19.55 (67.65)
Median (Q1,Q3)	4.00 (0.00,13.00)
Min,Max	0.00,752.00
Weight (kg)	
N (Missing)	7 (434)
Mean (SD)	53.20 (15.35)
Median (Q1,Q3)	50.00 (42.00,55.00)
Min,Max	37.90,85.00

### Distribution of adverse events at the SOC level

**Fig 2:**
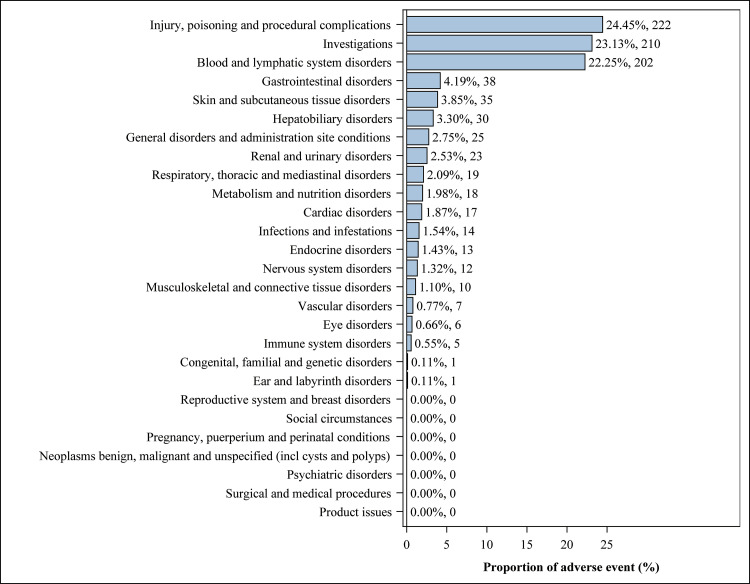
Proportion of adverse events by system organ class for toripalimab.

Adverse events linked to toripalimab cover 20 out of the 27 important Standardized Occurrence Categories (SOCs). Table 2 highlights significant results across different categories, with the five most prominent by proportion being Injury, Poisoning and Procedural Complications; Investigations; Disorders of the Blood and Lymphatic System; Gastrointestinal Disorders; and Disorders of the Skin and Subcutaneous Tissue. The intensity of signals for SOC-related adverse events associated with toripalimab in the FAERS database is illustrated in Table 2 and depicted in Fig 2.

**Table 2:** Signal strength of ADEs at the SOC level in FAERS database.

System Organ Class (SOC)	SOC Code	Case reports	ROR (95% CI)	PRR (95% CI)	IC (IC025)	EBGM (EBGM05)
Injury, poisoning and procedural complications	10022117	222	2.81 (2.41,3.27)	2.37 (2.11,2.65)	1.24 (1.02)	2.37 (2.03)
Investigations	10022891	210	4.59 (3.94,5.36)	3.76 (3.34,4.23)	1.91 (1.67)	3.76 (3.22)
Blood and lymphatic system disorders	10005329	202	16.69 (14.27,19.52)	13.20 (11.69,14.91)	3.72 (3.41)	13.20 (11.29)
Gastrointestinal disorders	10017947	38	0.47 (0.34,0.65)	0.49 (0.36,0.67)	−1.02 (−1.48)	0.49 (0.36)
Skin and subcutaneous tissue disorders	10040785	35	0.71 (0.50,0.99)	0.72 (0.52,0.99)	−0.48 (−0.96)	0.72 (0.51)
Hepatobiliary disorders	10019805	30	3.69 (2.57,5.31)	3.60 (2.53,5.12)	1.85 (1.20)	3.60 (2.50)
General disorders and administration site conditions	10018065	25	0.13 (0.09,0.20)	0.16 (0.11,0.23)	−2.66 (−3.19)	0.16 (0.11)
Renal and urinary disorders	10038359	23	1.34 (0.88,2.02)	1.33 (0.89,1.99)	0.41 (−0.21)	1.33 (0.88)
Respiratory, thoracic and mediastinal disorders	10038738	19	0.43 (0.27,0.68)	0.44 (0.29,0.69)	−1.17 (−1.78)	0.44 (0.28)
Metabolism and nutrition disorders	10027433	18	0.91 (0.57,1.45)	0.91 (0.58,1.44)	−0.13 (−0.79)	0.91 (0.57)
Cardiac disorders	10007541	17	0.70 (0.44,1.14)	0.71 (0.44,1.14)	−0.50 (−1.16)	0.71 (0.44)
Infections and infestations	10021881	14	0.28 (0.17,0.48)	0.29 (0.18,0.50)	−1.76 (−2.45)	0.29 (0.17)
Endocrine disorders	10014698	13	5.71 (3.30,9.88)	5.64 (3.29,9.68)	2.50 (1.31)	5.64 (3.26)
Nervous system disorders	10029205	12	0.14 (0.08,0.25)	0.16 (0.09,0.27)	−2.69 (−3.40)	0.16 (0.09)
Musculoskeletal and connective tissue disorders	10028395	10	0.20 (0.11,0.38)	0.21 (0.11,0.39)	−2.23 (−3.00)	0.21 (0.11)
Vascular disorders	10047065	7	0.36 (0.17,0.75)	0.36 (0.17,0.75)	−1.47 (−2.38)	0.36 (0.17)
Eye disorders	10015919	6	0.33 (0.15,0.73)	0.33 (0.15,0.74)	−1.59 (−2.54)	0.33 (0.15)
Immune system disorders	10021428	5	0.50 (0.21,1.20)	0.50 (0.21,1.20)	−1.00 (−2.06)	0.50 (0.21)
Ear and labyrinth disorders	10013993	1	0.25 (0.04,1.80)	0.25 (0.04,1.80)	−1.98 (−3.35)	0.25 (0.04)
Congenital, familial and genetic disorders	10010331	1	0.36 (0.05,2.58)	0.36 (0.05,2.58)	−1.46 (−2.95)	0.36 (0.05)

Note:ranked by case reports

### Distribution of adverse events at the PT level

Adverse events related to Toripalimab are categorized based on their frequency. Signals are identified when all of the following conditions are fulfilled: a ≥ 3, PRR ≥ 2, the lower limit of the 95% confidence interval (CI) for the ROR exceeds 1, IC025 is more than 0, and EBGM05 is greater than 2. This analysis seeks to uncover possible signals. Among the 20 most frequently reported adverse events are: off-label use, myelosuppression, reduced white blood cell count, lowered neutrophil count, decreased platelet count, diminished granulocyte count, liver damage, immune-mediated hepatic disorder, irregular liver function, hypothyroidism, medication errors, interstitial lung disease, mouth ulcers, erythematous rash, adrenal insufficiency, drug eruptions, electrolyte imbalances, reduced full blood count, myocarditis, papules, bicytopenia, drug-induced liver damage, and immune-mediated myocarditis (Table 3). Adverse events linked to Toripalimab are ranked according to their ROR signal strength. The five most prominent events are: Myelosuppression (n = 192, ROR 687.41), decreased granulocyte count (n = 11, ROR 515.72), immune-mediated hepatic disorder (n = 7, ROR 343.20), immune-mediated myocarditis (n = 3, ROR 214.68), and bicytopenia (n = 3, ROR 117.49). Comprehensive details about the adverse events associated with Toripalimab, classified by ROR signal strength at the PT level, can be found in Table 4.

**Table 3:** Signal strength of adverse events at the PT level ranked by ROR.

System Organ Class (SOC)	Preferred Term (PT)	Case reports	ROR (95% CI)	PRR (95% CI)	IC (IC025)	EBGM (EBGM05)
Blood and lymphatic system disorders	Myelosuppression	192	687.41 (585.86,806.57)	542.27 (477.91,615.29)	9.07 (6.92)	537.41 (458.01)
Investigations	Granulocyte count decreased	11	515.72 (283.86,936.96)	509.49 (282.45,919.01)	8.98 (2.71)	505.19 (278.07)
Hepatobiliary disorders	Immune-mediated hepatic disorder	7	343.20 (162.80,723.49)	340.56 (162.48,713.84)	8.40 (1.94)	338.64 (160.64)
Cardiac disorders	Immune-mediated myocarditis	3	214.68 (68.97,668.24)	213.97 (69.00,663.56)	7.74 (0.53)	213.22 (68.50)
Blood and lymphatic system disorders	Bicytopenia	3	117.49 (37.78,365.38)	117.11 (37.80,362.83)	6.87 (0.52)	116.88 (37.58)
Investigations	White blood cell count decreased	113	80.10 (65.76,97.55)	70.25 (59.11,83.49)	6.13 (5.16)	70.17 (57.62)
Investigations	Neutrophil count decreased	43	78.33 (57.66,106.42)	74.67 (55.77,99.99)	6.22 (4.36)	74.58 (54.90)
Skin and subcutaneous tissue disorders	Papule	3	30.80 (9.91,95.70)	30.70 (9.92,95.03)	4.94 (0.42)	30.68 (9.87)
Hepatobiliary disorders	Liver injury	9	29.92 (15.52,57.71)	29.64 (15.47,56.79)	4.89 (2.02)	29.62 (15.36)
Injury, poisoning and procedural complications	Off label use	210	23.24 (19.91,27.11)	18.09 (16.07,20.37)	4.18 (3.84)	18.09 (15.50)
Cardiac disorders	Myocarditis	3	20.28 (6.53,63.02)	20.22 (6.53,62.59)	4.34 (0.35)	20.21 (6.51)
Endocrine disorders	Adrenal insufficiency	3	19.27 (6.20,59.86)	19.21 (6.20,59.45)	4.26 (0.34)	19.20 (6.18)
Metabolism and nutrition disorders	Electrolyte imbalance	3	18.30 (5.89,56.86)	18.24 (5.89,56.47)	4.19 (0.33)	18.24 (5.87)
Endocrine disorders	Hypothyroidism	6	13.26 (5.94,29.59)	13.18 (5.93,29.25)	3.72 (1.17)	13.17 (5.90)
Gastrointestinal disorders	Mouth ulceration	4	13.18 (4.94,35.20)	13.13 (4.94,34.90)	3.71 (0.64)	13.12 (4.91)
Skin and subcutaneous tissue disorders	Drug eruption	3	12.06 (3.88,37.48)	12.03 (3.89,37.23)	3.59 (0.23)	12.03 (3.87)
Hepatobiliary disorders	Hepatic function abnormal	6	11.39 (5.10,25.43)	11.32 (5.10,25.14)	3.50 (1.10)	11.32 (5.07)
Investigations	Platelet count decreased	17	10.97 (6.79,17.72)	10.78 (6.73,17.27)	3.43 (2.12)	10.78 (6.67)
Investigations	Full blood count decreased	3	10.22 (3.29,31.74)	10.19 (3.29,31.52)	3.35 (0.18)	10.18 (3.28)
Hepatobiliary disorders	Drug-induced liver injury	3	7.57 (2.44,23.53)	7.55 (2.44,23.37)	2.92 (0.07)	7.55 (2.43)
Skin and subcutaneous tissue disorders	Rash erythematous	4	6.37 (2.39,17.01)	6.35 (2.39,16.88)	2.67 (0.32)	6.35 (2.38)
Injury, poisoning and procedural complications	Medication error	5	6.17 (2.56,14.86)	6.14 (2.56,14.72)	2.62 (0.54)	6.14 (2.55)
Respiratory, thoracic and mediastinal disorders	Interstitial lung disease	4	5.82 (2.18,15.53)	5.80 (2.18,15.41)	2.53 (0.27)	5.80 (2.17)

Note 1: Ranked by ROR

Note 2: Signals are detected when all the following criteria are met:a ≥ 3, PRR ≥ 2 and Chi-Square ≥ 4, lower limit of 95% CI of ROR > 1, IC025 > 0, EBGM05 > 2.

**Table 4:** Signal strength of adverse events at the PT level ranked by Reports.

System Organ Class (SOC)	Preferred Term (PT)	Case reports	ROR (95% CI)	PRR (95% CI)	IC (IC025)	EBGM (EBGM05)
Injury, poisoning and procedural complications	Off label use	210	23.24 (19.91,27.11)	18.09 (16.07,20.37)	4.18 (3.84)	18.09 (15.50)
Blood and lymphatic system disorders	Myelosuppression	192	687.41 (585.86,806.57)	542.27 (477.91,615.29)	9.07 (6.92)	537.41 (458.01)
Investigations	White blood cell count decreased	113	80.10 (65.76,97.55)	70.25 (59.11,83.49)	6.13 (5.16)	70.17 (57.62)
Investigations	Neutrophil count decreased	43	78.33 (57.66,106.42)	74.67 (55.77,99.99)	6.22 (4.36)	74.58 (54.90)
Investigations	Platelet count decreased	17	10.97 (6.79,17.72)	10.78 (6.73,17.27)	3.43 (2.12)	10.78 (6.67)
Investigations	Granulocyte count decreased	11	515.72 (283.86,936.96)	509.49 (282.45,919.01)	8.98 (2.71)	505.19 (278.07)
Hepatobiliary disorders	Liver injury	9	29.92 (15.52,57.71)	29.64 (15.47,56.79)	4.89 (2.02)	29.62 (15.36)
Hepatobiliary disorders	Immune-mediated hepatic disorder	7	343.20 (162.80,723.49)	340.56 (162.48,713.84)	8.40 (1.94)	338.64 (160.64)
Hepatobiliary disorders	Hepatic function abnormal	6	11.39 (5.10,25.43)	11.32 (5.10,25.14)	3.50 (1.10)	11.32 (5.07)
Endocrine disorders	Hypothyroidism	6	13.26 (5.94,29.59)	13.18 (5.93,29.25)	3.72 (1.17)	13.17 (5.90)
Injury, poisoning and procedural complications	Medication error	5	6.17 (2.56,14.86)	6.14 (2.56,14.72)	2.62 (0.54)	6.14 (2.55)
Respiratory, thoracic and mediastinal disorders	Interstitial lung disease	4	5.82 (2.18,15.53)	5.80 (2.18,15.41)	2.53 (0.27)	5.80 (2.17)
Gastrointestinal disorders	Mouth ulceration	4	13.18 (4.94,35.20)	13.13 (4.94,34.90)	3.71 (0.64)	13.12 (4.91)
Skin and subcutaneous tissue disorders	Rash erythematous	4	6.37 (2.39,17.01)	6.35 (2.39,16.88)	2.67 (0.32)	6.35 (2.38)
Endocrine disorders	Adrenal insufficiency	3	19.27 (6.20,59.86)	19.21 (6.20,59.45)	4.26 (0.34)	19.20 (6.18)
Skin and subcutaneous tissue disorders	Drug eruption	3	12.06 (3.88,37.48)	12.03 (3.89,37.23)	3.59 (0.23)	12.03 (3.87)
Metabolism and nutrition disorders	Electrolyte imbalance	3	18.30 (5.89,56.86)	18.24 (5.89,56.47)	4.19 (0.33)	18.24 (5.87)
Investigations	Full blood count decreased	3	10.22 (3.29,31.74)	10.19 (3.29,31.52)	3.35 (0.18)	10.18 (3.28)
Cardiac disorders	Myocarditis	3	20.28 (6.53,63.02)	20.22 (6.53,62.59)	4.34 (0.35)	20.21 (6.51)
Skin and subcutaneous tissue disorders	Papule	3	30.80 (9.91,95.70)	30.70 (9.92,95.03)	4.94 (0.42)	30.68 (9.87)
Blood and lymphatic system disorders	Bicytopenia	3	117.49 (37.78,365.38)	117.11 (37.80,362.83)	6.87 (0.52)	116.88 (37.58)
Hepatobiliary disorders	Drug-induced liver injury	3	7.57 (2.44,23.53)	7.55 (2.44,23.37)	2.92 (0.07)	7.55 (2.43)
Cardiac disorders	Immune-mediated myocarditis	3	214.68 (68.97,668.24)	213.97 (69.00,663.56)	7.74 (0.53)	213.22 (68.50)

Note1: Ranked by Reports

Note2: Signals are detected when all the following criteria are met:a ≥ 3, PRR ≥ 2 and Chi-Square ≥ 4, lower limit of 95% CI of ROR > 1, IC025 > 0, EBGM05 > 2.

### Time of occurrence of adverse events

**Fig 3:**
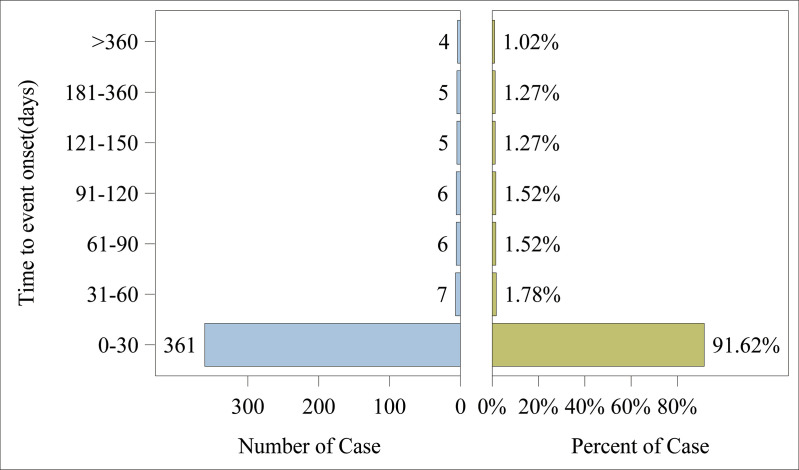
Time to onset of adverse events induced by toripalimab.

**Fig 4:**
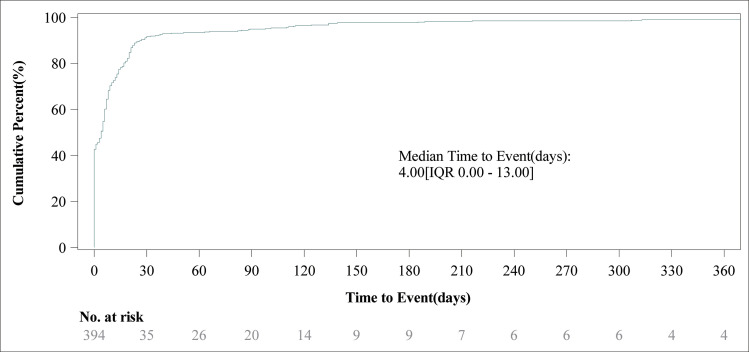
Cumulative incidence of adverse events related to toripalimab over time.

Adverse events associated with Toripalimab predominantly took place during the initial month of treatment (n = 361, 91.62%). Nevertheless, infrequent occurrences were noted at 2 months (n = 7, 1.78%), 3 months (n = 6, 1.52%), 4 months (n = 6, 1.52%), 5 months (n = 5, 1.27%), and from 6 to 12 months (n = 5, 1.27%), along with cases beyond 12 months (n = 4, 1.02%). The distribution of these events over time is depicted in Fig 3. Moreover, the median time until the event was recorded at 4 days (interquartile range [IQR] 0.00–13.00 days), and the cumulative incidence curve for adverse events is illustrated in Fig 4. An analysis using the Weibull distribution indicated the presence of an early failure mode, as shown in Table 5.

**Table 5:** Time-to-onset analysis using the Weibull distribution test.

		Weibull distribution				
Cases	TTO (days)	Scale parameter		Shape parameter		
n	median (IQR)	α	95% CI	β	95% CI	Failure type
394	4.00 (0.00,13.00)	23.88	19.56–29.15	0.70	0.64–0.76	Early failure

### Bibliometric analysis

**Fig 5:**
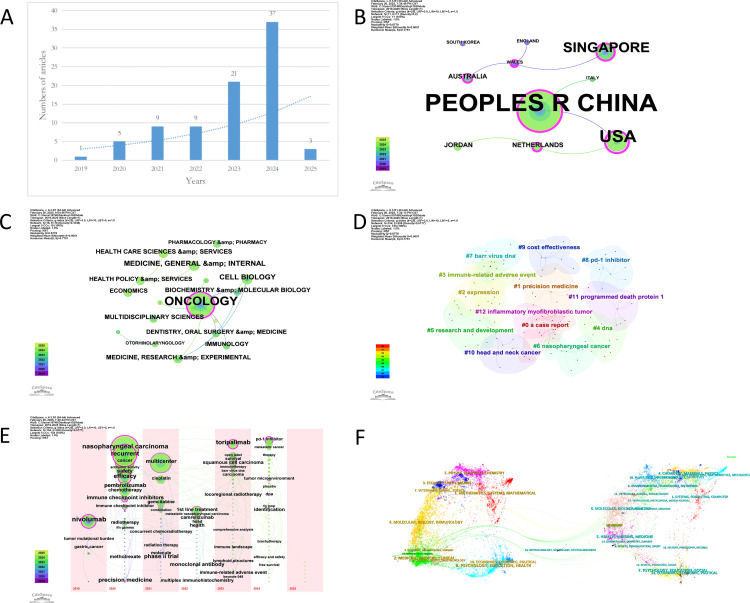
Bibliometric analysis.

This paper employs a multidimensional visualization approach to analyze the research trends related to Toripalimab and nasopharyngeal carcinoma. Since 2019, there has been a significant increase in the number of studies focused on the treatment of nasopharyngeal carcinoma with PD-1 inhibitors, which surged dramatically following the approval of Toripalimab by the U.S. FDA for this indication on October 27, 2023 (Fig 5A). This surge reflects the critical role that Toripalimab plays in the treatment landscape of nasopharyngeal carcinoma. China, the United States, and Singapore are the primary contributors in this field, with all three countries fostering the development of this area through close international cooperation (Fig 5B). Fig 5C illustrates the discipline co-occurrence network centered on Oncology, Cell Biology, and Medicine. The connections among these disciplines highlight their intersection and collaboration in the research of Toripalimab and nasopharyngeal carcinoma.Keyword cluster analysis reveals that the primary research topics in this field are Precision Medicine, Immune-Related Adverse Events, and Research and Development. Precision Medicine refers to personalized treatment based on a patient’s genetic information, environmental factors, and lifestyle. Immune-Related Adverse Events pertain to the study of potential side effects caused by immunotherapy, such as Toripalimab. Research and Development encompasses the processes involved in developing new drugs or treatment methods (Fig 5D). The time-zone diagram illustrates the transition in cancer treatment from Nivolumab to Toripalimab and from gastric cancer to nasopharyngeal carcinoma (Fig 5E). This trend highlights the significance of PD-1 inhibitor therapy in nasopharyngeal carcinoma. Furthermore, the analysis of journal distribution underscores the predominance of oncology and pharmacy journals, reinforcing the central role of Toripalimab in nasopharyngeal carcinoma (Fig 5F).

## Discussion

Among the immune checkpoint inhibitors used for the treatment of advanced cancers, toripalimab, a PD-1 inhibitor, has demonstrated significant improvements in the prognosis of cancers, including NPC [27]. Reports concerning AEs associated with toripalimab are limited, and its safety profile remains incompletely defined, which has consistently influenced its clinical application. To enhance the assessment of AEs related to toripalimab, we conducted an analysis utilizing the FAERS database. To our knowledge, this constitutes the most extensive and detailed examination documenting AEs linked to toripalimab via the FAERS database. This study affirmed previously acknowledged adverse reactions listed in the drug’s labeling for toripalimab. The noted reactions comprise common side effects such as nausea, fatigue, hypothyroidism, pneumonia, fever, vomiting, and decreased appetite. Furthermore, our analysis uncovered frequently noted grade 3 or 4 laboratory abnormalities (≥2%), which encompass neutropenia, lymphopenia, hemoglobin reduction, and thrombocytopenia. Additionally, immune-mediated adverse reactions were identified, which include liver injury, immune-mediated hepatic disorders, liver function abnormalities, renal failure, renal dysfunction, adrenal insufficiency, cardiac failure, and infusion-related reactions that manifest as itching and rashes. Moreover, we documented adverse events not listed on the label, such as off-label usage, medication errors, and interstitial lung disease. These findings underscore the necessity for diligent monitoring of the drug.In our investigation, off-label use was found to be the most frequently observed adverse reaction at the preferred term level. Potential factors contributing to off-label application may involve instances where the drug’s dosage exceeds the recommended levels specified on the label, use of the medication for indications not mentioned in the labeling, and administration for specific populations not addressed in the guidance, such as pediatric patients, older adults, or pregnant women. Moreover, off-label application may also entail modifications in the administration route or technique. A case report by Ning Zan et al. detailed a patient who received off-label immunotherapy (toripalimab) in conjunction with anti-angiogenic therapy, resulting in a sustained clinical response lasting over 25 months [28]. Additionally, Toripalimab is utilized off-label for the treatment of advanced cancer. This study aligns with the most recent reports regarding Toripalimab-related AEs, including decreased white blood cell count, decreased neutrophil count, and decreased platelet count [16,29,30]. Hematologic AEs, such as neutropenia, thrombocytopenia, and anemia, were prevalent, indicating the potential for myelosuppression or immune-mediated cytopenias [31]. It overlaps with the adverse reactions of other drugs [32,33]. Toripalimab acts as an immune checkpoint inhibitor, encouraging the immune system to target tumor cells; however, it may also inadvertently affect healthy organs and tissues, potentially leading to bone marrow suppression. In a particular study, all 33 patients who received toripalimab reported different levels of treatment-related adverse effects (TRAEs), including instances of bone marrow suppression. Common TRAEs encompassed myelosuppression, weight loss, and decreased appetite. Specifically, for grade 3 or higher adverse events, lymphopenia (82%), neutropenia (27%), and leukopenia (24%) were observed with increased frequency [34]. Frequent side effects encompass nausea, fatigue, underactive thyroid, pneumonia, fever, vomiting, and reduced appetite. These side effects highlight the significant stimulation of the immune system brought about by immune checkpoint inhibitors (ICIs), potentially resulting in widespread inflammation and immune dysfunction. Several clinical studies have found that hypothyroidism is a common adverse effect linked to toripalimab. For instance, in a phase II clinical trial of toripalimab for previously treated recurrent or metastatic nasopharyngeal carcinoma, the incidence of hypothyroidism was reported at 23.7% when administered at a dose of 3 mg/kg every two weeks [15]. Furthermore, a multicenter randomized phase III trial involving 408 patients with nasopharyngeal carcinoma demonstrated that adverse events, including hypothyroidism, occurred more frequently in the toripalimab group compared to the placebo group (30.8% vs. 16.8%) [14]. Our research is consistent with earlier investigations, emphasizing the necessity of vigilant observation and management of this negative response during the administration of toripalimab for treating nasopharyngeal carcinoma. Additionally, our study revealed adverse effects not currently included in drug labeling, such as interstitial lung disease. Although these adverse reactions were not recorded on the drug label, a patient in the Phase Ib/II clinical trial NCT02915432 reported a treatment-related death due to interstitial lung disease while receiving toripalimab for chemotherapy-refractory gastric cancer [35]. The exact mechanism underlying this condition may be linked to the way toripalimab enhances the anti-tumor immune response by promoting the differentiation of lymphocytes and increasing levels of cytokines and autoantibodies. This process could lead to increased infiltration of immune cells and cytokines in lung tissue undergoing radiotherapy, potentially causing damage to both cancerous and healthy lung cells. As a result, healthcare professionals must closely monitor patients with nasopharyngeal carcinoma who are at risk of developing interstitial lung disease. There was a notable trend observed in adverse events related to toripalimab, particularly regarding immune-mediated hepatic disorders (n = 7, ROR 343.20, PRR 340.56, IC 8.40, EBGM 338.64) and immune-mediated myocarditis (n = 3, ROR 214.68, PRR 213.97, IC 7.74, EBGM 213.22). These findings further underscore the vulnerability of the liver and heart to immune-mediated injury [36,37]. ICIs stimulate anti-tumor immune responses; however, they may also induce autoimmunity and other immunopathological mechanisms, which can ultimately result in immune-related adverse events [38]. Adrenal insufficiency (n = 3, ROR 19.21) is a potential adverse reaction signal outside of the drug label. Many studies have shown the importance of this adverse reaction and its organic close association with other systemic diseases, and a large number of studies have used bibliometrics to demonstrate the importance of this adverse reaction [39–41].

This investigation also conducted a temporal examination of adverse events and utilized the Weibull distribution to forecast the timing of such occurrences, thereby aiding in the development of an effective schedule for overseeing drug-related adverse reactions. The findings highlight the critical necessity of vigilant monitoring, especially during the first month of toripalimab therapy. This initial monitoring period is crucial for identifying and addressing possible adverse effects, thus enhancing patient safety and therapeutic outcomes.

This study faces several limitations. First, the FAERS database, which depends on voluntary reports from consumers, healthcare providers, and pharmacists, may inherently contain gaps or inaccuracies in its information; for instance, it does not include data regarding patients’ exposure to medications. Second, the dataset analyzed in this study was limited, indicating the need for a more comprehensive dataset to verify our findings. However, our research focused on the drug itself and detailed its specific indications for use, which enhances the specificity of the results. Third, a substantial amount of the data is sourced from China, which might introduce reporting biases. Future studies should strive to incorporate data from multiple countries to enhance generalizability. Finally, while the causality analysis successfully identified positive signals for adverse events, it did not establish a definitive causal relationship between toripalimab and these events. Long-term prospective studies are essential to confirm the potential adverse effects highlighted in this study.

## Conclusion

This study provides a comprehensive analysis of the safety profile of Toripalimab, utilizing data from the FAERS database to deliver crucial safety insights concerning the drug’s application in clinical settings. Our findings not only confirm the previously documented adverse effects of Toripalimab but also uncover further potential risks. These results serve as a vital reference for healthcare professionals, including clinicians and pharmacists, aimed at improving medication management and addressing safety concerns associated with Toripalimab.

## Supporting information

S1 Table Two-by-two contingency table for disproportionality analyses. (DOCX)

S2 Table Four major algorithms used for signal detection. (DOCX)
